# Cardio-Metabolic Disease Risks and Their Associations with Circulating 25-Hydroxyvitamin D and Omega-3 Levels in South Asian and White Canadians

**DOI:** 10.1371/journal.pone.0147648

**Published:** 2016-01-25

**Authors:** Chao-Wu Xiao, Carla M. Wood, Eleonora Swist, Reiko Nagasaka, Kurtis Sarafin, Claude Gagnon, Lois Fernandez, Sylvie Faucher, Hong-Xing Wu, Laura Kenney, Walisundera M. N. Ratnayake

**Affiliations:** 1 Nutrition Research Division, Bureau of Nutritional Sciences, Health Canada, Ottawa, Canada; 2 Food Chemistry and Functional Nutrition, Department of Food Science and Technology, Graduate School of Marine Science and Technology, 5–7, Konan 4, Minato, Tokyo, Japan; 3 Centre for Biologics Evaluation, Biologics and Genetic Therapies Directorate, Health Canada, Ottawa, Canada; 4 Food and Nutrition Science Program, Department of Chemistry, Carleton University, Ottawa, Canada; 5 Biostatistics and Modelling Division, Bureau of Food Surveillance and Science Integration, Health Canada, Ottawa, Canada; Medical University Innsbruck, AUSTRIA

## Abstract

**Objectives:**

This study compared cardio-metabolic disease risk factors and their associations with serum vitamin D and omega-3 status in South Asian (SAC) and White Canadians (WC) living in Canada’s capital region.

**Methods:**

Fasting blood samples were taken from 235 SAC and 279 WC aged 20 to 79 years in Ottawa, and 22 risk factors were measured.

**Results:**

SAC men and women had significantly higher fasting glucose, insulin, homeostasis model assessment for insulin resistance (HOMA-IR), apolipoprotein B (ApoB), ratios of total (TC) to HDL cholesterol (HDLC) and ApoB to ApoA1, leptin, E-selectin, P-selectin, ICAM-1 and omega-3 (p < 0.05), but lower HDLC, ApoA1, vitamin D levels than WC (p < 0.05). SAC women had higher CRP and VEGF than WC women. Adequate (50–74.9 nmol/L) or optimal (≥ 75 nmol/L) levels of 25(OH)D were associated with lower BMI, glucose, insulin, HOMA-IR, TG, TC, low density lipoprotein cholesterol (LDLC), ApoB/ApoA1 ratio, CRP, leptin, and higher HDLC, ApoA1, omega-3 index, L-selectin levels in WC, but not in SAC. Intermediate (>4%-<8%) or high (≥ 8%) levels of omega-3 indices were related to lower E-selectin, P-selectin, ICAM-1 and higher HDLC, 25(OH)D levels in WC, but not in SAC. The BMIs of ≤ 25 kg/m^2^ were related to lower LDLC, ApoB, VEGF, creatinine and higher 25(OH)D in WC, but not in SAC.

**Conclusions:**

The associations of vitamin D, omega-3 status, BMI and risk factors were more profound in the WC than SAC. Compared to WC, vitamin D status and omega-3 index may not be good predictive risk factors for the prevalence of CVD and diabetes in SAC.

## Introduction

Cardiovascular (CVD) and diabetic diseases are the leading causes of death worldwide [1]. The prevalence of CVD is expected to increase, predominantly because of increasing sedentary lifestyles and prevalence of obesity and diabetes mellitus [2]. In recent years, an increasing number of studies have shown that South Asians are more prone to cardio-metabolic diseases, such as CVD and type 2 diabetes [3]. Compared to other ethnic groups, the incidence and mortality of CVD in South Asians are 50% to 300% higher, and the ages to develop coronary heart disease are 5 to 10 years earlier [3].

Canadians of South Asian origin are one of the largest non-European groups and accounted for 4.1% of Canadian population in 2006 [4]. South Asian Canadians (SAC) also have higher risk for heart disease, stroke, premature CVD [5,6], hypertension and incidence of type 2 diabetes [7,8]. Conventional risk factors such as smoking, hypertension, diabetes, or high cholesterol [9,10] cannot explain the excess CVD risk in South Asians. Compared to other ethnic groups, SAC aged 35 to 75 yrs had the highest prevalence of the carotid atherosclerosis, which is associated with increased incidence of CVD, increased prevalence of glucose intolerance, higher total (TC) and LDL cholesterol (LDLC), triglycerides (TG) and lower HDL cholesterol (HDLC) as well as greater abnormalities of novel risk factors including higher levels of fibrinogen, homocysteine, lipoprotein (a) and plasminogen activator inhibitor-1 [5].

Vitamin D status is associated with many health outcomes such as bone density, certain cancers, autoimmune conditions, and CVD [11]. A study in young Canadians aged 20 to 29 yrs showed that individuals of South Asian descent had significantly lower plasma 25(OH)D, higher glucose, fasting insulin, LDLC and lower HDLC compared to white Canadians (WC) [12]. Vitamin D deficiency was associated with higher insulin, and homeostasis model assessment for insulin resistance (HOMA-IR) among Caucasians, but Vitamin D status was not associated with any of those biomarkers measured in SAC [12]. However, these ethnicity-specific relations of vitamin D and cardio-metabolic disease risks have not been established in other age groups of Canadians.

Accumulated evidence showed that regular consumption of fish or long chain omega-3 fatty acids (FA), especially eicosapentaenoic (EPA) and docosahexaenoic (DHA) acids, has beneficial effects on CVD such as reducing arrhythmias, endothelial dysfunction, blood TG, and inflammation [13–17]. Both dietary intake and red blood cell (RBC) membrane levels of EPA + DHA are associated with a reduced risk of primary cardiac arrest [18]. Omega-3 index, the sum of EPA and DHA as a percentage of total FA in RBC membranes, has been proposed as a biomarker of omega-3 FA intake and of CVD risk [19–21]. Omega-3 indices of ≤4%, 4–8% and ≥8% have been shown to be associated with high, intermediate, and low risk levels, respectively [20]. However, the omega-3 status in the Canadian population has not been assessed, and particularly the association of omega-3 status and cardio-metabolic disease risks in different ethnic groups remain unclear.

Objectives of this study were, (a) to determine the vitamin D, omega-3 status, and cardio-metabolic disease risk factors in SAC and WC living in the National Capital Region of Canada; (b) to assess the associations of vitamin D, omega-3 status and cardio-metabolic disease risk factors as well as whether vitamin D levels and omega-3 index can be considered as risk factors for the prevalence of CVD in SAC compared to WC living in the same geographical location.

## Methods

### Subjects and Sample collection

The study was approved by Health Canada and the Public Health Agency of Canada Research Ethics Board (REB 2010–0043) and all participants provided written informed consent. Participants were recruited through advertisements in the National Capital Region of Canada. These advertisements were published in local French and English newspapers as well as in Sri Lankan, Indian, Pakistani, Nepalese, and Bangladeshi community newspapers, websites, sports arenas and community centers. Electronic news broadcasts from Health Canada, Agriculture and Agri-Food Canada and Canadian Food Inspection Agency also posted advertisements. Data on ethnicity, gender, and age were collected from information provided in the initial enrollment form and in the medication questionnaire. The exclusion criteria were age and ethnicity. Only South Asian and European descent (i.e., white Canadians or Caucasians) volunteers between the ages of 20 and 79 years were recruited. Participant ethnicity was assessed based on responses to a questionnaire pertaining to the individual’s birthplace and ancestry. SAC are people with origin in India, Pakistan, Bangladesh, Sri Lanka, Nepal, and Bhutan [22]. White Canadians are people with European ancestry.

Eligible participants attended one of the clinics held on nine different dates between April 14^th^ and May 6^th^, 2012. Among the 739 volunteers enrolled, 649 (308 SAC and 341 WC) attended the clinics. Expenses for the participants travelling to the clinics were paid as needed. Blood samples were collected from the antecubital vein of the subjects after an overnight fast (>10 hrs) into one 2-ml Serum Separator and one 2-ml EDTA tube at the end of winter (April~May), 2012. All samples were processed within 3 hrs and packed RBC isolated. Samples were kept at -80°C until analysis. Height (m) and body weight (kg) were measured and the body mass index (BMI) was calculated as (body weight in kg)/(height in m)^2^. To be comparable, the same cut-off points, BMI ≤ 25 kg/m^2^, 25.1–29.9 kg/m^2^, and ≥ 30 kg/m^2^ were used in both ethnic groups. Data of the participants with diabetes or using cholesterol lowering medication based on the information provided in the medication questionnaire were excluded (73 SAC and 62 WC) in the analysis of this study.

### Measurement of blood insulin, leptin, glucose, creatinine, FGF-23 and lipid profiles

Plasma insulin, leptin (Alpco Diagnostics, Salem, NH) and serum fibroblast growth factor-23 (FGF-23)(RayBiotech, Norcross, GA) levels were measured using Enzyme-Linked Immunosorbent Assay kits. Serum glucose levels were determined by a colorimetric assay [23]. Serum TC, HDLC [24] and TG [25] contents were measured by enzymatic assays. LDLC levels were calculated using the equation: LDLC = TC–HDLC—TG/2.22. Serum apolipoprotein A1 (ApoA1) and B (ApoB) levels were measured through immunochemical reactions using specific antisera against human ApoA1 and ApoB. Serum creatinine levels were determined using the enzymatic isotope dilution mass spectrometry-standardized two-point rate method. All the measurements were conducted in the Ortho Clinical Diagnostics Vitros 5.1FS Analyzer (Ortho Clinical Diagnostics, Rochester, NY). Fasting glucose level < 5.6 mmol/L was considered normal, 5.6–6.9 mmol/L impaired, and ≥ 7 mmol/L diabetic according to the guidelines for definition, diagnosis and classification of Type 2 diabetes published by the International Diabetes Federation[26]. Low HDLC was defined as < 1 mmol/L for men and < 1.3 mmol/L for women, and high ratio of TC to HDLC as >4. HOMA-IR was calculated as fasting insulin (μIU/ml) x fasting glucose (mmol/L)/22.5, and HOMA-IR > 3.0 indicates a likely significant insulin resistance [27].

### Determination of serum vitamin D

Serum vitamin D levels were measured as 25-hydroxyvitamin D [25(OH)D] concentrations using the Diasorin LIAISON® TOTAL 25(OH)D chemiluminesence assay (DiasorinInc, Stillwater, MN, USA) according to manufacturer’s instruction of use. The inter- and intra-assay variability ranged from 6.4–9.4% and 4.9–6.1%, respectively. Individual vitamin D status were categorized as deficiency (< 30 nmol/L), insufficiency (30–49.9 nmol/L), adequacy (50–74.9 nmol/L) and optimum (≥75 nmol/L) according to the recommendations of the Institute of Medicine [28], the Endocrine Society [29], and the Canadian Osteoporosis Society [30]. Since the number of subjects with 25(OH)D level < 30 nmol/L in WC was too small [9 (3.2%)], we combined deficiency with insufficiency to 25(OH)D < 50 nmol/L.

### RBC omega-3 indices

The FA composition in the RBC samples was analyzed within 4 wks of storage (-80°C) by gas chromatography (GC) according to the procedure of Pottala et al. [31]. The analyses were performed using an Agilent 6890N network GC equipped with a SP-2560, 100 m x 0.25 mm internal diameter, 0.2 μm film thickness flexible fused silica capillary column (Supelco, Bellefonte, PA). Omega-3 index is defined as the sum of EPA and DHA as a percentage of total FA in RBC membranes [19]. SAC and WC were grouped by their omega-3 indices into ≤ 4%, 4–8% and ≥ 8% [20], respectively.

### Plasma C-reactive protein (CRP), vitamin D binding protein (VDBP) and vascular inflammatory factors

Plasma CRP and VDBP were analyzed using in-house developed bead assays (Luminex Corp., Toronto, Canada). CRP was detected using antibody pairs (R&D Systems, Minneapolis, USA) and recombinant CRP as the calibrator. VDBP was captured on bead using polyclonal anti-human VDBP and detected with a biotinylated anti-VDBP monoclonal antibody. A mixed human VDBP was used as the calibrator (Cedarlane, Burlington,Canada). CRP and VDBP assays were run in single-plex assays. Plasma E-selectin, L-selectin, P-selectin, intercellular adhesion molecule 1 (ICAM-1) and vascular endothelial growth factor (VEGF) levels were analyzed using a bead-based multiplex immunoassay (BD Biosciences, Mississauga, Canada).

### Statistical Analysis

Results were expressed as means ± SEM. All data were assessed for equality of variance, and the variables with skewed distribution were logarithmically transformed prior to statistical analysis. The data presented were untransformed for ease of interpretation. The differences in the risk factors or biomarkers for the cardio-metabolic diseases between WC and SAC were examined using multivariable linear regression models and adjusted by covariates, age and gender. Exploratory analyses in the models including an interaction term for either vitamin D status or omega-3 index or BMI x ethnicity, as well as age and gender were conducted before the associations between the cardio-metabolic risk factors and vitamin D status or omega-3 index or BMI across ethnic groups were examined. Subsequently, analyses were stratified by ethnicity and adjusted for age and gender. The models were tested for collinearity of covariates including age, 25(OH)D, ethnicity, and gender using the variance inflation factor, which confirmed no significant collinearity. The independent association of ethnicity with prevalence of selected serum risk factors relative to a threshold indicating at-risk status was examined using multiple logistic regression models and adjusted by covariates, gender and age. A probability of *p* < 0.05 was considered to be significant. All analyses were performed by using SAS EG (version 5.1, SAS Institute Inc, Cary, NC).

## Results

### Age and BMI of the study subjects

Eligible participates in this study included 119 SAC women, 116 SAC men, 197 WC women, and 82 WC men. The average ages and BMIs were not significantly different between WC and SAC (p > 0.05, **Table 1**). The percentages of subjects with BMIs of 25.1–29.9 and ≥30 in both ethnic groups were not different (p > 0.05, **Table 2**).

**Table 1:** Risk factors for cardiovascular diseases and diabetes in White (WC) and South Asian Canadians (SAC) living in Ottawa*.

	Women		Men			p value	
	WC	SAC	WC	SAC	Sex	Ethnic	Sex x Ethnic
Number (n)	197	119	82	116			
Age (years)	46.76 ± 0.93	44.89 ± 1.13	47.44 ± 1.55	46.54 ± 1.34	ns	ns	ns
BMI (kg/m^2^)	25.47 ± 0.39	25.51 ± 0.39	26.44 ± 0.44	25.96 ± 0.33	ns	ns	ns
Glucose (mmol/L)	4.45 ± 0.03 a	4.64 ± 0.06 b	4.61 ± 0.06 a	5.02 ± 0.10 b	<0.0001	<0.0001	ns
Insulin (mIU/L)	5.66 ± 0.35 a	8.39 ± 0.67 b	8.15 ± 1.99 a	9.02 ± 0.57 b	ns	<0.0001	ns
HOMA-IR	1.15 ± 0.08 a	1.78 ± 0.15 b	1.70 ± 0.39 a	2.09 ± 0.16 b	0.0172	<0.0001	ns
TG (mmol/L)	1.08 ± 0.05	1.18 ± 0.06	1.32 ± 0.10	1.49 ± 0.104	0.0003	ns	ns
TC (mmol/L)	5.18 ± 0.08	5.07 ± 0.09	5.11 ± 0.11	5.21 ± 0.08	ns	ns	ns
HDLC (mmol/L)	1.66 ± 0.03 a	1.42 ± 0.03 b	1.28 ± 0.04 a	1.18 ± 0.03 b	<0.0001	<0.0001	0.0500
LDLC (mmol/L)	3.06 ± 0.07	3.11 ± 0.08	4.43 ± 1.20	4.22 ± 0.84	0.0297	ns	ns
TC:HDLC	3.27 ± 0.07 a	3.68 ± 0.08 b	4.23 ± 0.14 a	4.67 ± 0.12 b	<0.0001	<0.0001	ns
ApoA1 (g/L)	1.62 ± 0.02 a	1.43 ± 0.02 b	1.41 ± 0.03 a	1.28 ± 0.02 b	<0.0001	<0.0001	ns
ApoB (g/L)	1.01 ± 0.02 a	1.08 ± 0.03 b	1.06 ± 0.03 a	1.16 ± 0.02 b	0.0203	0.0014	ns
ApoB:ApoA1	0.64 ± 0.02 a	0.78 ± 0.02 b	0.78 ± 0.03 a	0.93 ± 0.03 b	<0.0001	<0.0001	ns
CRP (mg/L)	1.29 ± 0.10 a	1.58 ± 0.14 b	0.96 ± 0.12	1.11 ± 0.10	0.0198	0.0147	ns
Leptin (μg/L)	15.66 ± 0.96 a	23.87 ± 1.24 b	6.31 ± 0.77 a	9.36 ± 0.86 b	ns	<0.0001	ns
25(OH)D (nmol/L)	74.21 ± 2.23 a	54.84 ± 2.61 b	69.10 ± 3.84 a	44.85 ± 1.63 b	0.0047	<0.0001	ns
Omega-3 index	5.96 ± 0.12 a	6.62 ± 0.16 b	5.83 ± 0.22 a	6.44 ± 0.15 b	ns	0.0001	ns
VDBP (mg/L)	234.63 ± 4.29	237.37 ± 4.44	226.62 ± 3.95	220.47 ± 3.66	0.0072	ns	ns
E-selectin (μg/L)	7.62 ± 0.31 a	9.66 ± 0.51 b	9.53 ± 0.63 a	12.36 ± 0.65 b	<0.0001	<0.0001	ns
L-selectin (mg/L)	0.61 ± 0.02	0.58 ± 0.02	0.57 ± 0.03	0.56 ± 0.02	ns	ns	ns
p-selectin (μg/L)	49.11 ± 1.82 a	56.61 ± 2.80 b	53.89 ± 3.02 a	67.06 ± 3.23 b	0.0052	0.0002	ns
ICAM-1 (μg/L)	116.93 ± 2.52 a	125.56 ± 3.24 b	113.09 ± 3.63 a	129.25 ± 3.43 b	ns	0.0002	ns
VEGF (ng/L)	21.71 ± 0.96 a	32.08 ± 3.54 b	21.72 ± 1.83	22.82 ± 1.70	0.0314	0.0077	0.0309
CREA (μmol/L)	79.46 ± 0.81	76.17 ± 1.21	100.09 ± 1.64	100.78 ± 1.48	<0.0001	ns	ns
FGF-23 (μg/L)	1.85 ± 0.31	1.27 ± 0.29	2.95 ± 0.55	2.18 ± 0.56	0.0202	ns	ns

Values are presented as mean ± SEM. Means with different letters in the same row and sex differ (p < 0.05). ApoA1 = apolipoprotein A1, ApoB = apolipoprotein B, BMI = body mass index, CRP = C-reactive protein, CREA = creatinine, FGF-23 = fibroblast growth factor-23, HDLC = high density lipoprotein cholesterol, HOMA-IR = homeostasis model assessment for insulin resistance, ICAM-1 = intercellular adhesion molecule 1, LDLC = low density lipoprotein cholesterol, ns = not significant, TC = total cholesterol, TG = triglyceride, 25(OH)D = 25-hydroxyvitamin D, VDBP = vitamin D binding protein, VEGF = vascular endothelial growth factor.

*The average differences in the cardio-metabolic risk factors or biomarkers between SAC and WC were analyzed by multivariable linear regression models and adjusted for age and gender.

**Table 2:** Comparison of BMI, serum glucose, HOMA-IR, HDLC levels and ratio of TC to HDLC in White (WC) and South Asian Canadians (SAC) living in Ottawa^a^.

	WC	SAC	p value
BMI			
≤ 25	136^b^(48.9)^c^	102 (43.8)	
25.1–29.9	95 (34.2)	102 (43.8)	
≥ 30	47 (16.9)	29 (12.4)	0.0656
Subtotal	278	233	
Glucose (mmol/L)			
< 5.6	269 (96.4)	211 (89.8)	
5.6–6.9	9 (3.2)	20 (8.5)	
≥ 7	1 (0.4)	4 (1.7)	0.0096
Subtotal	279	235	
HOMA-IR			
> 3	17 (6.1)	37 (15.7)	
< 3	262 (93.9)	198 (84.3)	0.0004
Subtotal	279	235	
TG (mmol/L)			
< 1.7	235 (84.2)	180 (76.6)	
≥ 1.7	44 (15.8)	55 (23.4)	0.0300
Subtotal	279	235	
HDLC (mmol/L)			
≥1 for men≥1.3 for women	228 (81.7)	161 (68.5)	
<1 for men<1.3 for women	51(18.3)	74 (31.5)	0.0001
Subtotal	279	235	
TC:HDL			
≤ 4	196 (70.3)	125 (53.2)	
> 4	83 (29.7)	110 (46.8)	<0.0001
Subtotal	279	235	

BMI = body mass index, HDLC = high density lipoprotein cholesterol, HOMA-IR = homeostasis model assessment for insulin resistance, TC = total cholesterol, TG = triglyceride.

^a^The independent association of ethnicity with prevalence of serum disease risk factors relative to a threshold indicating at-risk status was examined by multiple logistic regression models and adjusted by covariates, age and gender

^b^Number of subjects under each category

^c^percentage of the subtotal.

### Biomarkers for Type 2 diabetes and kidney function

Serum glucose levels in both SAC women and men were significantly higher than in WC counterparts (p < 0.0001, Table 1). Percentages of SAC with fasting blood glucose reaching the levels for glucose intolerance (5.6–6.9 mmol/L, 8.5% vs 3.2%) or diabetes (≥ 7 mmol/L, 1.7% vs 0.4%) were much higher than those of the WC (p < 0.0096, Table 2). Plasma insulin levels (p = 0.0128) and the HOMA-IR scores (p = 0.0016) in SAC were markedly higher than in WC (Table 1). More SAC than WC showed insulin resistance (HOMA-IR > 3.0, 15.7% vs 6.1%, p = 0.0004, Table 2). Serum creatinine levels were significantly higher in the men than in the women (p < 0.0001), and not different between the ethnic groups (p > 0.05).

### Risk factors for CVD

Serum TG, TC and LDLC levels were not different in the two ethnic groups (p > 0.05, Table 1), whereas the HDLC levels in SAC were significantly lower than in the WC (p < 0.0001). Overall, there were higher percentages of SAC with unhealthy HDLC levels (< 1 mmol/L for men, or < 1.3 mmol/L for women, p < 0.0001, Table 2). Compared to the WC, the ratios of TC to HDLC in the SAC were significantly higher in both sexes (p < 0.0001). Percentage of the SAC with ratio of TC to HDLC > 4 (46.8%) was significantly higher than in WC (29.7%) (p< 0.0001, Table 2).

Plasma CRP level was significantly higher in SAC women than in the WC women (p = 0.0147, Table 1), but was not different between SAC and WC men. Leptin levels in both SAC sexes were markedly higher than in WC (p < 0.0001). The SAC had lower serum ApoA1 and higher ApoB levels than WC, leading to remarkably higher ApoB/ApoA1 ratio in the SAC (p < 0.0001, Table 1). Serum FGF-23 levels were markedly higher in the men than in the women (p = 0.0202), but not different between the ethnic groups (p > 0.05).

### Vitamin D, Omega-3 index, VDBP, and Vascular adhesion molecules

Compared to WC, SAC had significantly lower serum 25(OH)D levels, but higher RBC omega-3 indices (p < 0.0001, Table 1). Plasma E-selectin, p-selectin, and ICAM-1 concentrations in SAC were markedly higher than in WC (p < 0.001). VEGF content in SAC women was significantly higher than in WC men (p < 0.01). Plasma VDBP and L-selectin levels were not different between the ethnic groups (p > 0.05).

### Vitamin D status and risk factors for CVD and diabetes

The SAC with adequate (50–74.9 nmol/L) or optimal (≥ 75 nmol/L) levels of serum 25(OH)D had lower ratios of serum TC to HDLC (p < 0.05) and higher levels of VDBP (p < 0.01) than those with inadequate or deficient (<50 nmol/L) levels of 25(OH)D (**Table 3**). The SAC with optimal level of serum 25(OH)D had significantly lower serum ApoB (P < 0.05) than those with deficient or inadequate levels of 25(OH)D.

**Table 3:** Association of Vitamin D Status and Risk Factors for Cardiovascular Diseases and Diabetes in White (WC) and South Asian Canadians (SAC) living in Ottawa*.

	WC				SAC			
	25(OH)D (nmol/L)				25(OH)D (nmol/L)			
	< 50	50–74.9	≥ 75	p value	< 50	50–74.9	≥ 75	p value
Number (%)	54 (19.4)	121 (43.4)	104 (37.2)		108 (53.7)	70 (34.8)	23 (11.5)	
BMI (kg/m^2^)	28.0 ± 0.82 a	26.0 ± 0.45 b	24.3 ± 0.44 c	<0.0001	26.0 ± 0.35	25.2 ± 0.42	25.8 ± 0.78	ns
Glucose (mmol/L)	4.6 ± 0.08 a	4.5 ± 0.04 b	4.4 ± 0.04 b	0.0073	4.9 ± 0.09	4.8 ± 0.07	4.9 ± 0.16	ns
Insulin (mIU/L)	8.0 ± 0.94 a	6.8 ± 1.35 b	5.1 ± 0.43 b	0.0061	8.9 ± 0.48	8.4 ± 1.02	8.5 ± 1.45	ns
HOMA-IR	1.7 ± 0.22 a	1.4 ± 0.26 b	1.0 ± 0.09 b	0.0028	2.0 ± 0.14	1.8 ± 0.22	1.9 ± 0.36	ns
TG (mmol/L)	1.4 ± 0.12 a	1.1 ± 0.06 b	1.0 ± 0.08 b	0.0007	1.4 ± 0.09	1.2 ± 0.07	1.4 ± 0.09	ns
TC (mmol/L)	5.5 ± 0.19 a	5.1 ± 0.10 b	5.0 ± 0.10 b	0.0003	5.2 ± 0.08	5.2 ± 0.11	4.8 ± 0.20	ns
HDLC (mmol/L)	1.4 ± 0.05 a	1.5 ± 0.04 b	1.6 ± 0.04 b	0.0064	1.3 ± 0.03	1.4 ± 0.04	1.3 ± 0.08	ns
LDLC (mmol/L)	5.3 ± 1.81 a	3.1 ± 0.08 b	2.9 ± 0.09 b	<0.0001	4.0 ± 0.69	3.2 ± 0.10	2.9 ± 0.19	ns
TC:HDLC	4.2 ± 0.17 a	3.6 ± 0.11 b	3.2 ± 0.09 c	<0.0001	4.4 ± 0.10 a	3.9 ± 0.14 b	3.9 ± 0.26 b	0.0444
ApoA1 (g/L)	1.4 ± 0.03 a	1.5 ± 0.03 b	1.6 ± 0.03 b	0.0089	1.3 ± 0.02	1.4 ± 0.03	1.4 ± 0.07	ns
ApoB (g/L)	1.1 ± 0.04 a	1.0 ± 0.03 b	1.0 ± 0.03 b	0.0004	1.1 ± 0.02 a	1.1 ± 0.03 ab	1.0 ± 0.05 b	0.0492
ApoB/ApoA1	0.8 ± 0.03 a	0.7 ± 0.02 b	0.6 ± 0.02 c	<0.0001	0.9 ± 0.02	0.8 ± 0.03	0.78± 0.06	ns
CRP (mg/L)	1.6 ± 0.24 a	1.1 ± 0.10 b	1.1 ± 0.11 b	0.0048	1.4 ± 0.11	1.4 ± 0.17	0.9 ± 0.13	ns
Leptin (μg/L)	15.5 ± 1.82 a	13.1 ± 1.12 ab	11.4 ± 1.24 b	0.0263	15.5 ± 1.13	17.6 ± 1.42	21.6 ± 3.95	ns
Omega-3 index	5.2 ± 0.14 a	5.8 ± 0.13 b	6.5 ± 0.21 c	<0.0001	6.5 ± 0.14	6.6 ± 0.20	6.4 ± 0.42	ns
VDBP (mg/L)	235.7 ± 7.37	231.1 ± 4.44	232.1 ± 6.23	ns	224.7 ± 3.47 a	235.9 ± 5.77 b	235.3 ± 11.22 b	0.0020
E-selectin (μg/L)	9.4 ± 0.64 a	7.7 ± 0.44 b	8.1 ± 0.48 b	0.0500	11.3 ± 0.53	11.3 ± 0.79	8.2 ± 1.18	ns
L-selectin (mg/L)	0.5 ± 0.03 a	0.6 ± 0.02 b	0.6 ± 0.02 b	0.0015	0.6 ± 0.02	0.6 ± 0.03	0.5 ± 0.05	ns
p-selectin (μg/L)	48.9 ± 3.45	51.2 ± 2.60	50.6 ± 2.30	ns	61.5 ± 2.82	62.7 ± 3.97	60.1 ± 6.22	ns
ICAM-1 (μg/L)	115.7 ± 4.24	113.6 ± 3.45	118.5 ± 3.18	ns	127.2 ± 3.16	128.2 ± 4.22	125.6 ± 6.19	ns
VEGF (ng/L)	21.6 ± 1.72	22.2 ± 1.38	21.2 ± 1.43	ns	26.2 ± 2.80	28.6 ± 3.40	33.1 ± 4.83	ns
CREA (μmol/L)	82.24 ± 2.03	86.87 ± 1.51	85.65 ± 1.43	ns	88.23 ± 1.52	87.30 ± 2.40	91.35 ± 4.65	ns
FGF-23 (μg/L)	1.80 ± 0.46	2.44 ± 0.51	2.05 ± 0.38	ns	1.26 ± 0.23	2.17 ± 0.63	3.20 ± 2.13	ns

Values are presented as mean ± SEM. Means with different letters in the same row and ethnic group differ (p < 0.05). ApoA1 = apolipoprotein A1, ApoB = apolipoprotein B, BMI = body mass index, CRP = C-reactive protein, CREA = creatinine, FGF-23 = fibroblast growth factor-23, HDLC = high density lipoprotein cholesterol, HOMA-IR = homeostasis model assessment for insulin resistance, ICAM-1 = intercellular adhesion molecule 1, LDLC = low density lipoprotein cholesterol, ns = not significant, TC = total cholesterol, TG = triglyceride, 25(OH)D = 25-hydroxyvitamin D, VDBP = vitamin D binding protein, VEGF = vascular endothelial growth factor.

*Exploratory analyses in the models including an interaction term for vitamin D status x ethnicity, as well as age and gender, were conducted before the associations between cardio-metabolic risk factors and vitamin D status across ethnic groups were examined by multivariable linear regression models. The analyses were stratified by ethnicity and adjusted for age and gender.

The WC with optimal levels of 25(OH)D had the lowest BMI, ratios of TC:HDLC, ApoB/ApoA1 and highest omega-3 index, and those with adequate levels of 25(OH)D had markedly lower BMI, ratios of TC:HDLC, ApoB/ApoA1 and higher omega-3 index than those with inadequate or deficient levels of 25(OH)D. The WC with optimal or adequate levels of 25(OH)D had significantly lower plasma glucose, insulin, HOMA-IR, TG, TC, LDLC, ApoB, CRP, E-selectin, L-selectin and higher ApoA1 than those with inadequate or deficient levels of 25(OH)D. Additionally, the WC with optimal level of 25(OH)D had lower leptin than those with inadequate and/or deficient levels of 25(OH)D (Table 3). However, most of these associations did not exist in SAC. Serum creatinine and FGF-23 levels were not different in both WC and SAC with different 25(OH)D status (p > 0.05, Table 3).

### Omega-3 indices and risk factors for CVD and diabetes

Omega-3 status was not associated with any of the diabetic biomarkers (glucose, insulin, HOMA-IR), serum creatinine and FGF-23 levels in either ethnic group (**Table 4**). The WC with high level (≥8%) of omega-3 indices had significantly lower TG (p = 0.0077), and higher 25(OH)D (<0.0001) levels than those with intermediate (>4%—<8%) or low (≤4%) omega-3 indices, and had higher HDLC and lower CRP than those with intermediate levels of omega-3. Moreover, the WC with intermediate levels of omega-3 indices had lower E-selectin, p-selectin and ICAM-1 levels than those with low omega-3 (p < 0.01). None of these beneficial effects of omega-3 were observed in the SAC.

**Table 4:** Association of Omega-3 Status and Risk Factors for Cardiovascular Diseases and Diabetes in White (WC) and South Asian Canadians (SAC) living in Ottawa*.

		WC				SAC		
	Omega-3 index				Omega-3 index			
	≤ 4%	>4%-<8%	≥ 8%	p value	≤ 4%	>4%-<8%	≥ 8%	p value
Number (%)	26 (9.3)	225 (80.7)	28 (10.0)		15 (6.4)	172 (73.2)	48 (20.4)	
BMI (kg/m^2^)	25.6 ± 0.97 ab	26.0 ± 0.34 a	23.8 ± 0.97 b	0.0461	25.9 ± 0.84	25.6 ± 0.29	26.1 ± 0.65	ns
Glucose (mmol/L)	4.5 ± 0.10	4.5 ± 0.03	4.5 ± 0.09	ns	4.7 ± 0.11	4.8 ± 0.06	5.0 ± 0.18	ns
Insulin (mIU/L)	6.8 ± 1.18	6.6 ± 0.77	4.2 ± 0.57	ns	8.3 ± 1.21	8.4 ± 0.42	10.0 ± 1.49	ns
HOMA-IR	1.4 ± 0.25	1.4 ± 0.15	0.9 ± 0.14	ns	1.7 ± 0.25	1.9 ± 0.11	2.3 ± 0.34	ns
TG (mmol/L)	1.4 ± 0.13 a	1.2 ± 0.05 a	0.9 ± 0.08 b	0.0077	1.5 ± 0.18	1.4 ± 0.07	1.2 ± 0.09	ns
TC (mmol/L)	5.3 ± 0.23	5.1 ± 0.07	5.4 ± 0.23	ns	5.4 ± 0.25	5.1 ± 0.07	5.2 ± 0.15	ns
HDLC (mmol/L)	1.6 ± 0.11 ab	1.5 ± 0.03 a	1.8 ± 0.09 b	0.0337	1.3 ± 0.07	1.3 ± 0.03	1.3 ± 0.04	ns
LDLC (mmol/L)	3.1 ± 0.19	3.1 ± 0.06	3.2 ± 0.19	ns	3.4 ± 0.23	3.2 ± 0.06	3.4 ± 0.13	ns
TC:HDLC	3.8 ± 0.26	3.6 ± 0.08	3.1 ± 0.15	ns	4.2 ± 0.28	4.1 ± 0.10	4.3 ± 0.17	ns
ApoA1 (g/L)	1.6 ± 0.07	1.5 ± 0.02	1.6 ± 0.05	ns	1.4 ± 0.05	1.4 ± 0.02	1.3 ± 0.03	ns
ApoB (g/L)	1.0 ± 0.06	1.0 ± 0.02	1.0 ± 0.07	ns	1.1 ± 0.05	1.1 ± 0.02	1.2 ± 0.05	ns
ApoB/ApoA1	0.7 ± 0.05	0.7 ± 0.02	0.6 ± 0.04	ns	0.8 ± 0.04	0.8 ± 0.02	0.9 ± 0.04	ns
CRP (mg/L)	1.3 ± 0.29 ab	1.2 ± 0.09 a	0.8 ± 0.17 b	0.0413	1.2 ± 0.26	1.3 ± 0.10	1.5 ± 0.23	ns
Leptin (μg/L)	10.3 ± 1.93	13.3 ± 0.84	12.1 ± 2.89	ns	20.8 ± 3.67	16.0 ± 1.08	17.9 ± 1.68	ns
25(OH)D (nmol/L)	72.5 ± 10.4 a	69.3 ± 1.60 a	100.4 ± 9.49 b	<0.0001	47.4 ± 7.13	50.3 ± 1.96	49.4 ± 2.46	ns
VDBP (mg/L)	223.6 ± 9.83	232.7 ± 3.76	236.8 ± 7.19	ns	226.0 ± 11.65	230.0 ± 3.63	226.8 ± 4.97	ns
E-selectin (μg/L)	11.0 ± 0.95 a	7.8 ± 0.31 b	8.3 ± 1.03 b	0.0060	9.6 ± 1.28	10.6 ± 0.49	12.6 ± 0.96	ns
L-selectin (mg/L)	0.6 ± 0.06	0.6 ± 0.02	0.7 ± 0.05	ns	0.5 ± 0.10	0.6 ± 0.02	0.5 ± 0.04	ns
p-selectin (μg/L)	64.9 ± 6.50 a	48.4 ± 1.64 b	53.6 ± 5.14 ab	0.0060	64.7 ± 11.80	63.8 ± 2.51	53.4 ± 4.00	ns
ICAM-1 (μg/L)	136.9 ± 8.47 a	112.2 ± 2.06 b	125.2 ± 8.51 ab	0.0009	131.5 ± 11.52	127.6 ± 2.81	125.1 ± 4.48	ns
VEGF (ng/L)	25.7 ± 2.41	21.1 ± 1.00	23.4 ± 2.48	ns	52.6 ± 20.17	26.1 ± 1.93	24.7 ± 2.24	ns
CREA (μmol/L)	86.65 ± 3.91	85.77 ± 1.02	82.5 ± 2.73	ns	82.73 ± 3.60	89.71 ± 1.50	84.83 ± 2.59	ns
FGF-23 (μg/L)	2.98 ± 1.78	2.08 ± 0.26	2.15 ± 0.63	ns	0.77 ± 0.30	1.85 ± 0.40	1.56 ± 0.50	ns

Values are presented as mean ± SEM. Means with different letters in the same row and ethnic group differ (p < 0.05). ApoA1 = apolipoprotein A1, ApoB = apolipoprotein B, BMI = body mass index, CRP = C-reactive protein, CREA = creatinine, FGF-23 = fibroblast growth factor-23, HDLC = high density lipoprotein cholesterol, HOMA-IR = homeostasis model assessment for insulin resistance, ICAM-1 = intercellular adhesion molecule 1, LDLC = low density lipoprotein cholesterol, ns = not significant, TC = total cholesterol, TG = triglyceride, 25(OH)D = 25-hydroxyvitamin D, VDBP = vitamin D binding protein, VEGF = vascular endothelial growth factor. Omega-3 index = [(eicosapentaemoic acid + docosahexaenoic acid)/total fatty acid] *100.

*Exploratory analyses in the models including an interaction term for omega-3 index x ethnicity, as well as age and gender, were conducted before the associations between cardio-metabolic risk factors and omega-3 index across ethnic groups were examined by multivariable linear regression models. The analyses were stratified by ethnicity and adjusted for age and gender.

### BMI and risk factors for CVD and diabetes

The WC with BMIs ≤ 25 kg/m^2^ had significantly lower LDLC, ApoB, 25(OH)D, creatinine and VEGF levels than those with 25.1–29.9 kg/m^2^ or ≥ 30 kg/m^2^ BMIs (**Table 5**). However, these associations were not significant in SAC (p > 0.05). In the SAC with BMIs ≤ 25 kg/m^2^, plasma insulin, HOMA-IR, TG, HDLC, LDLC, ratios of TC:HDLC, and leptin levels were similar to those in the WC with BMIs of 25.1–29.9 kg/m^2^, and the ratio of ApoB/ApoA1, 25(OH)D, E-selectin, and ICAM-1 levels close to those in the WC with BMIs ≥ 30 kg/m^2^. The association between serum FGF-23 levels and BMI were not significant in both ethnic groups.

**Table 5:** Association of BMI and risk factors for Cardiovascular Diseases and Diabetes in White (WC) and South Asian Canadians (SAC) living in Ottawa*.

		WC				SAC		
	BMI (kg/m^2^)				BMI (kg/m^2^)			
	≤ 25	25.1–29.9	≥ 30	p value	≤ 25	25.1–29.9	≥ 30	p value
Number (%)	136 (48.7)	95 (34.1)	48 (17.2)		102 (43.4)	102 (43.4)	31 (13.2)	
Glucose (mmol/L)	4.4 ± 0.04 a	4.5 ± 0.04 a	4.8 ± 0.08 b	<0.0001	4.7 ± 0.08 a	4.9 ± 0.10 ab	5.1 ± 0.18 b	0.0365
Insulin (mIU/L)	4.1 ± 0.34 a	7.4 ± 1.67 b	10.7 ± 1.08 b	0.0015	7.2 ± 0.71 a	8.5 ± 0.56 a	14.1 ± 1.15 b	<0.0001
HOMA-IR	0.8 ± 0.07 a	1.5 ± 0.32 b	2.4 ± 0.27 c	0.0001	1.5 ± 0.15 a	1.9 ± 0.16 a	3.3 ± 0.32 b	<0.0001
TG (mmol/L)	1.0 ± 0.06 a	1.1 ± 0.06 a	1.7 ± 0.14 c	<0.0001	1.1 ± 0.05 a	1.5 ± 0.11 b	1.5 ± 0.13 b	0.0187
TC (mmol/L)	5.0 ± 0.09	5.3 ± 0.12	5.4 ± 0.15	ns	5.1 ± 0.08	5.2 ± 0.10	5.2 ± 0.18	ns
HDLC (mmol/L)	1.7 ± 0.04 a	1.5 ± 0.04 b	1.3 ± 0.05 c	<0.0001	1.4 ± 0.03 a	1.2 ± 0.03 b	1.3 ± 0.06 ab	0.0044
LDLC (mmol/L)	2.9 ± 0.08 a	3.3 ± 0.10 b	3.4 ± 0.14 b	0.0272	3.2 ± 0.07	3.3 ± 0.09	3.3 ± 0.17	ns
TC:HDLC	3.1 ± 0.07 a	3.7 ± 0.12 b	4.5 ± 0.20 c	<0.0001	3.9 ± 0.11 a	4.5 ± 0.13 b	4.2 ± 0.20 ab	0.0243
ApoA1 (g/L)	1.6 ± 0.03 a	1.5 ± 0.03 ab	1.4 ± 0.05 b	0.0013	1.4 ± 0.03 a	1.3 ± 0.02 b	1.4 ± 0.03 ab	0.0165
ApoB (g/L)	1.0 ± 0.03 a	1.1 ± 0.03 ab	1.1 ± 0.04 b	0.0424	1.1 ± 0.03	1.1 ± 0.03	1.1 ± 0.05	ns
ApoB/ApoA1	0.6 ± 0.02 a	0.7 ± 0.03 b	0.8 ± 0.04 c	<0.0001	0.8 ± 0.02 a	0.9 ± 0.03 b	0.8 ± 0.05 ab	0.0101
CRP (mg/L)	0.8 ± 0.08 a	1.3 ± 0.14 b	2.2 ± 0.20 c	<0.0001	1.0 ± 0.11 a	1.4 ± 0.12 b	2.4 ± 0.29 c	<0.0001
Leptin (μg/L)	7.7 ± 0.72 a	13.1 ± 1.00 b	27.2 ± 2.36 c	<0.0001	12.5 ± 0.95 a	17.0 ± 1.40 b	29.5 ± 2.81 c	<0.0001
25(OH)D (nmol/L)	79.0 ± 2.92 a	71.5 ± 3.37 a	57.3 ± 2.78 b	0.0001	50.1 ± 1.93	51.2 ± 2.92	44.9 ± 3.30	ns
Omega-3 index	6.0 ± 0.17	6.0 ± 0.15	5.4 ± 0.20	ns	6.8 ± 0.17	6.3 ± 0.16	6.6 ± 0.31	ns
VDBP (mg/L)	234.9 ± 4.79	231.7 ± 5.82	225.9 ± 6.35	ns	232.5 ± 4.53	222.9 ± 3.82	238.2 ± 10.53	ns
E-selectin (μg/L)	7.2 ± 0.33 a	9.0 ± 0.62 b	9.4 ± 0.60 b	0.0224	9.3 ± 0.50 a	12.0 ± 0.68 b	13.4 ± 1.41 b	0.0012
L-selectin (μg/L)	633.8 ± 21.9	552.1 ± 26.1	599.7 ± 31.3	ns	596.1 ± 25.1	543.4 ± 25.7	559.0 ± 45.5	ns
p-selectin (μg/L)	50.9 ± 2.32	48.5 ± 2.48	53.4 ± 4.00	ns	58.0 ± 2.94	64.3 ± 3.39	65.6 ± 7.10	ns
ICAM-1 (μg/L)	116.7 ± 3.10 ab	110.2 ± 3.32 a	124.4 ± 4.91 b	0.0386	120.1 ± 2.90 a	129.5 ± 3.74 ab	145.6 ± 8.14 b	0.0014
VEGF (ng/L)	19.4 ± 1.15 a	23.9 ± 1.53 b	23.9 ± 2.27 ab	0.0378	23.1 ± 2.03	32.0 ± 3.87	27.8 ± 5.03	ns
CREA (μmol/L)	83.14 ± 1.08 a	88.45 ± 1.83 b	86.89 ± 2.59 ab	0.0317	87.2 ± 1.80	89.64 ± 1.87	87.62 ± 4.41	ns
FGF-23 (μg/L)	1.96 ± 0.31	2.40 ± 0.43	2.32 ± 1.06	ns	1.68 ± 0.52	1.99 ± 0.48	1.00 ± 0.39	ns

Values are presented as mean ± SEM. Means with different letters in the same row and ethnic group differ (p < 0.05). ApoA1 = apolipoprotein A1, ApoB = apolipoprotein B, BMI = body mass index, CRP = C-reactive protein, CREA = creatinine, FGF-23 = fibroblast growth factor-23, HDLC = high density lipoprotein cholesterol, HOMA-IR = homeostasis model assessment for insulin resistance, ICAM-1 = intercellular adhesion molecule 1, LDLC = low density lipoprotein cholesterol, ns = not significant, TC = total cholesterol, TG = triglyceride, 25(OH)D = 25-hydroxyvitamin D, VDBP = vitamin D binding protein, VEGF = vascular endothelial growth factor. BMI cut-off points: ≤ 25 kg/m^2^, 25.1–29.9 kg/m^2^, and ≥30 kg/m^2^.

*Exploratory analyses in the models including an interaction term for BMI x ethnicity, as well as age and gender, were conducted before the associations between cardio-metabolic risk factors and BMI across ethnic groups were examined by multivariable linear regression models. The analyses were stratified by ethnicity and adjusted for age and gender.

## Discussion

This cross-sectional study has analyzed a comprehensive set of risk factors and biomarkers for cardio-metabolic diseases in SAC relative to WC aged 20 to 79 yrs. SAC living in the Canada’s Capital region had significantly higher risks for metabolic diseases such as CVD and type 2 diabetes than the WC living in the same geographical region as reflected by the observed differences in multiple cardio-metabolic disease risk factors. These include remarkably higher fasting glucose, insulin, HOMA-IR score, ratio of TC to HDLC, ApoB, ratio of ApoB to ApoA1, leptin, E-selectin, P-selectin, ICAM-1, and lower HDLC, ApoA1 levels in both SAC women and men, and higher CRP and VEGF in SAC women. The prevalence of insulin resistance, unhealthy levels of HDLC, and ratio of TC to HDLC were markedly higher in SAC than in WC. Serum 25(OH)D levels in SAC men and women were significantly lower than in WC, whereas the RBC omega-3 indices in SAC were higher. These results further showed the ethnic-specific differences in cardio-metabolic disease risks and status of vitamin D and omega-3.

ICAM-1, E-selectin and p-selectin are essential for the firm adhesion of leukocytes to vascular endothelium [32,33] and important in the pathogenesis of CVD [34–36]. Increased serum levels of these molecules are suggested to be independent risk factors for atherosclerosis and CVD. Blood ICAM-1 and E-selectin levels are associated with the established CVD risk factors such as smoking, waist-hip ratio, blood pressure, HDL and total cholesterol [37], and are related to the earliest stages of atherosclerosis in obese, hypertensive and children with diabetes [38]. These factors may contribute to CVD through their inflammatory effects on the vascular endothelium [37]. Serum levels of ICAM-1, E-selectin, P-selectin, L-selectin were higher in patients with type 2 diabetes, and associated with glycemic control, disturbances of lipid metabolism, obesity and insulin resistance. Adhesion molecules play important roles in pathogenesis of microangiopathy and macroangiopathy in type 2 diabetes [39]. The plasma E-selectin level was negatively correlated with insulin sensitivity in the obese women [40].

The present study also showed that the SAC had remarkably higher fasting glucose, insulin and HOMA-IR score than WC. The conventional risk factors, TG, TC and LDLC, were not different between the two ethnic groups. However, the levels of HDLC were lower, and ratio of ApoB to ApoA1 and leptin were higher in SAC than in WC. Although the underlying mechanism(s) by which SAC have higher risks for CVD and diabetes than WC remain unclear, both genetic and non-genetic factors may play a role. For example, South Asian men aged at 40–70 yrs had ~20% lower maximal oxygen uptake values than Europeans during moderate intensity physical activity, which is suggested to be a key factor associated with the excess insulin resistance and fasting glycaemia in middle-aged South Asian compared with European men [41]. For a given age and BMI, South Asian men need greater levels of physical activity to achieve a similar cardio-metabolic risk profile to that observed in European men [42]. The BMI and waist circumference cut-points required in South Asian men to have a similar cardio-metabolic risk profiles to European men at conventional obesity thresholds (BMI 30 kg/m^2^ and waist circumference 102 cm) were lower [43,44]. Additionally, duration of residence in the USA appears to be an important determinant for CVD risk factors among South Asian immigrants [22]. Lifestyle factors such as lack of exercise, excess caloric intake, excess sugar intake and abdominal obesity have been suggested as possible factors contributing CVD in South Asians [45,46]. Creatinine is a reliable indicator of kidney function. Increased creatinine level may suggest impaired kidney function or kidney disease [47]. The serum levels of creatinine in this study were remarkably higher in men than in women, but were not different between the ethnic groups.

Both sexes of SAC in this study showed significantly lower serum 25(OH)D levels compared to WC. Overall, 53.7% SAC vs 19.4% WC were vitamin D deficient or inadequate (25(OH)D < 50 nmol/L). Our study has shown that vitamin D status is associated with many risk factors in the WC including BMI, fasting glucose, insulin, HOMA-IR, TG, TC, HDLC, LDLC, ratios of TC to HDLC, and ApoB to ApoA1, ApoA1, ApoB, CRP, leptin, omega-3 indices, E-selectin and L-selectin. However, most of these associations were not present in SAC except for the ratio of TC:HDLC and ApoB. This is in line with a study showing the association of vitamin D deficiency with insulin resistance (higher insulin and HOMA-IR) in Caucasians and East Asians, but not in South Asians [12]. Although the high risk for CVD and diabetes in South Asians remains unexplained, the unique phenotype of having greater visceral adipose tissue (VAT) in South Asians than Europeans even at the same BMI may be one of the contributing factors, which has been shown to mediate risk factors including TC, LDL-C, TC/HDL-C, glucose, and diastolic blood pressure in men, and in HDL-C, TG, TC/HDL-C, and HOMA in women [48]. FGF-23 is a newly discovered glycoprotein hormone that regulates phosphorus and vitamin D metabolism. Increased FGF-23 levels have been associated with higher risks for CVD [49,50]. However, the present study showed that the serum FGF-23 levels did not differ between ethnic groups, and that their association with 25(OH)D status, omega-3 indices and BMI were not significant.

Regular consumption of fish or long chain omega-3 FA, especially EPA and DHA, has beneficial effects on CVD, such as reducing arrhythmias, endothelial dysfunction, blood triglycerides, and inflammation[13–17]. The recent FAO/WHO dietary guidelines recommend consumption of at least 0.25 g and up to 2.0 g of EPA + DHA per day for the prevention of coronary heart disease and other degenerative diseases of aging [51]. The present study was the first to assess the omega-3 status and its association with risk factors for CVD and diabetes in different ethnic groups of the Canadian population. The results showed that the omega-3 indices in SAC were markedly higher than those of the WC. This appears to be inconsistent with the high risk for CVD and diabetes in South Asians. Our further analysis suggested that the higher RBC omega-3 indices are associated with decreased risk for these diseases only in WC, including lower TG, CRP, E-selectin, P-selectin, ICAM-1 and higher HDLC and 25(OH)D levels, but unrelated to any of the risk factors measured in SAC. Interestingly, the levels of another set of RBC FA including 18:2 n-6, 18:3 n-3 and 20:4 n-6 were closely associated with the risk factors (TG, TC, LDL, ratio of TC to HDLC, ApoB and the ratio of ApoB to ApoA1) in SAC (data not shown). This suggests that the cardio-protective function of omega-3 FA may be ethnicity-dependent and not universal. Dietary recommendation of EPA+DHA intake may not benefit certain subpopulation such as South Asians.

Our results also showed that BMI is closely associated with most of the cardio-metabolic disease risk factors measured (16 out of the remaining 21) in the WC, but with less (12 of the remaining 21 factors) in SAC. Interestingly, the levels of many risk factors for CVD and diabetes in the SAC with BMI ≤ 25 were at the same levels as those in the WC with overweight (25.1–29.9) or obese (≥ 30) BMI. This is in line with the discovery that at the same BMI, Asians had higher risk of developing type 2 diabetes [52], hypertension and CVD [53–55] than Whites. Although the underlying mechanisms involved in these ethnic differences remain unclear, higher body fat in Asians might be one of the possible explanations. Particularly, South Asians have especially high levels of body fat and are more prone to developing abdominal obesity, which may contribute to their high risk of cardio-metabolic diseases [56].

A main strength of this study is that a set of comprehensive risk factors or biomarkers for CVD and type 2 diabetes were included in the analysis. The omega-3 index and its association with cardio-metabolic disease risk were first compared within different Canadian ethnic groups. The limitation of this study is that cross-sectional design may lead to biased selection of subjects since the volunteers who usually participate in scientific studies will have higher education and socio-economic class than the general population. The results of the study may not fully represent those of the Canadian population. Another limitation is that the health-related activities such physical exercises, cigarette smoking and dietary practices were not adjusted. A further limitation is that the categories of vitamin D deficiency (25(OH)D <30 nmol/L) and inadequacy (50 nmol/L >25(OH)D ≥ 30) were not separated.

In conclusion, the present study has shown that the SAC aged 20 to 79 yrs living in the Canada’s capital region exhibited a significantly higher risk for cardio-metabolic diseases than the WC living in the same region. Serum 25(OH)D levels in SAC were significantly lower than in WC. The associations of vitamin D, omega-3 status, BMI and risk factors were more profound in the WC than SAC. Although RBC omega-3 index in SAC was higher, it appears to be unrelated to any of the risk factors. Overall, many of the cardio-metabolic benefits of vitamin D, omega-3 and normal BMI shown in WC were absent in SAC.

## Abbreviations

25(OH)D: 25-hydroxyvitamin D
ApoA1: apolipoprotein A1
ApoB: apolipoprotein B
BMI: body mass index
CRP: C-reactive protein
DHA: docosahexaenoic acid
EPA: eicosapentaemoic acid
FGF-23: fibroblast growth factor 23
HDLC: high density lipoprotein cholesterol
HOMA-IR: homeostasis model assessment for insulin resistance
ICAM-1: intercellular adhesion molecule 1
LDLC: low density lipoprotein cholesterol
SAC: South Asian Canadian
TG: triglyceride
VDBP: vitamin D binding protein
VEGF: vascular endothelial growth factor
WC: White Canadian
